# 1632. Bivalent RSV Prefusion F-Based Subunit Vaccine Generates High Neutralizing Titers in Older Adults

**DOI:** 10.1093/ofid/ofad500.1466

**Published:** 2023-11-27

**Authors:** Yasushi Fukushima, Jose F Cardona, Agnieszka Zareba, Qin Jiang, Daniel P Eiras, Michael Patton, Kumar Ilangovan, Elliot N DeHaan, Tarek Mikati, Elena Kalinina, David Cooper, Kenneth Koury, Annaliesa S Anderson, Kena A Swanson, William C Gruber, Alejandra C Gurtman, Beate Schmoele-Thoma

**Affiliations:** Fukuwa clinic, Chuo-ku, Tokyo, Japan; Indago Research & Health Center, Hialeah, Florida; Pfizer, Pearl RIver, New York; Pfizer, Pearl RIver, New York; Pfizer, Inc., Pearl River, New York; Pfizer, Vaccine Research and Development, Hurley, England, United Kingdom; Pfizer, Vaccine Research and Development, Hurley, England, United Kingdom; Pfizer, Pearl RIver, New York; 3. Pfizer, Inc., Vaccine Research & Development, Pearl River, New York; Pfizer, Pearl RIver, New York; Pfizer, Pearl RIver, New York; Pfizer, Pearl RIver, New York; Pfizer, Pearl RIver, New York; Pfizer, Pearl RIver, New York; Pfizer, Pearl RIver, New York; Pfizer, Pearl RIver, New York; Pfizer, Pearl RIver, New York

## Abstract

**Background:**

Respiratory syncytial virus (RSV) is an important cause of lower respiratory tract illness (LRTI) in older adults. The search for an effective RSV vaccine was hindered by poor functional immunogenicity of early vaccine candidates that did not have F antigens stabilized in the prefusion state. RSVpreF, Pfizer’s bivalent stabilized prefusion F subunit vaccine candidate containing preF antigens against two major RSV subgroups (A and B), elicited robust neutralizing responses in phase 1/2 studies.

**Methods:**

The RSV Vaccine Efficacy Study in Older Adults Immunized Against RSV Disease (RENOIR) is a phase 3, multicenter, randomized, double-blinded, placebo-controlled study evaluating vaccine efficacy (VE) to prevent LRTI in adults ≥60 years of age over two RSV seasons (NCT05035212). Participants were randomly assigned (1:1 ratio) to receive a single intramuscular injection of RSVpreF at a dose of 120μg or placebo. The study demonstrated a VE of 88.9% for LRTI-RSV with ≥3 symptoms at the end of the first RSV season. A secondary objective was to describe the immune responses induced by RSVpreF following vaccination. The immunogenicity subset included 1150 participants enrolled from sites in the United States and Japan.

**Results:**

Here we report the immunogenicity results one month after RSVpreF vaccination. The neutralization titer geometric mean fold rises (GMFRs) ranged from 11.6 to 12.7 for RSV A and B, respectively, and GMFRs for combined RSV A/B neutralizing responses ranged from 12.0 to 13.0 for subgroups stratified by age (60-69, 70-79 and 80+ years). RSV A/B GMFRs in participants with prespecified chronic conditions were similar to or higher than those without, with 11.4, 13.0 and 14.8, respectively, for subgroups stratified based on none, at least one prespecified chronic medical condition or at least one chronic cardiopulmonary condition.

RSV Neutralizing GMTs Before and 1 Month Postvaccination 1 and Subgroup Analysis

**Figure:**
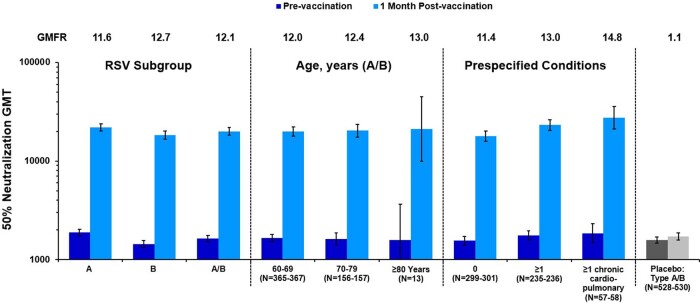

**Conclusion:**

High RSV neutralizing titers were observed one month after RSVpreF vaccination in adults 60 years and older, consistent with prior phase 1/2 studies. The study further shows similarly robust responses across age subgroups and baseline chronic conditions. Although there is no established correlate of protection, these high antibody responses corresponded with high RSVpreF VE in the first RSV season.

**Disclosures:**

**Agnieszka Zareba, MD PhD**, Pfizer: Employee|Pfizer: Stocks/Bonds|Pfizer: Stocks/Bonds **Qin Jiang, PhD**, Pfizer: Employee|Pfizer: Employee|Pfizer: Stocks/Bonds|Pfizer: Stocks/Bonds **Daniel P. Eiras, MD, MPH**, Pfizer, Inc.: Stocks/Bonds **Michael Patton, B.Sc.**, Pfizer Inc.: Employee|Pfizer Inc.: Stocks/Bonds **Kumar Ilangovan, MD, MSPH, MMCi**, Pfizer, Inc.: Employee|Pfizer, Inc.: Stocks/Bonds **Elliot N. DeHaan, MD**, Pfizer: Employee|Pfizer: Stocks/Bonds **Tarek Mikati, MD,MPH**, Pfizer: Stocks/Bonds **Elena Kalinina, PhD**, Pfizer: Pfizer employee|Pfizer: Stocks/Bonds **David Cooper, PhD**, Pfizer, Inc.: Stocks/Bonds **Kenneth Koury, PhD**, Pfizer: Employee|Pfizer: Stocks/Bonds **Annaliesa S. Anderson, PhD**, Pfizer: Employee|Pfizer: Stocks/Bonds **Kena A. Swanson, Ph.D.**, Pfizer: Employee|Pfizer: Stocks/Bonds **William C. Gruber, MD**, Pfizer, Inc.: Employee|Pfizer, Inc.: Stocks/Bonds **Alejandra C. Gurtman, M.D.**, Pfizer: Employee|Pfizer: Stocks/Bonds **Beate Schmoele-Thoma, MD**, Pfizer: Stocks/Bonds

